# Foliar microbiota confers deeper color of fermented cigar wrapper under additional fermented bacteria

**DOI:** 10.1186/s40643-025-00921-5

**Published:** 2025-07-22

**Authors:** Qian Wu, Xuanshuang Duan, Dapeng Tang, Rui Wang, Fuqiang Li, Jun Tan, Honghua Chen, Shixue Zheng

**Affiliations:** 1 National Key Laboratory of Agricultural Microbiology, College of Life Science and Technology, Huazhong Agricultural University, Wuhan, Hubei 430070 P. R. China; 2 Enshi Branch, Hubei Tobacco Company, Enshi, Hubei 445000 P. R. China; 3 Tobacco Research Institute of Hubei Province, Wuhan, Hubei 430033 P. R. China

**Keywords:** Cigar wrapper color, Fermentation, Microbial community structure, Enzyme activity, Metabolome

## Abstract

Microorganisms play an indispensable role in the fermentation of cigar tobacco such as improvement of flavor, aroma and quality. However, little is known about additional microorganism impact on shift of the color of cigar wrappers. Here, three bacterial strains that could alter the wrapper’s color were isolated and screened from cigar tobacco leaves (CTLs) at different stages of fermentation. The three strains were identified to be *Sphingomonas parapaucimobilis* DS1, *Kosakonia cowanii* DS101, and *Comamonas thiooxydans* DS309 based on the genome sequences. The overall color difference (∆E) of CTLs was significantly deepened with the addition of those three strains under upscaled stack fermentation, respectively (*p* ≤ 0.05), showing the same fundamental quality of cigar wrapper. The ascorbic acid oxidase activity was significantly decreased and cigarillo polyphenol oxidase activity was significantly increased added with the three bacteria compared to the control (*p* ≤ 0.05). Moreover, the exogenous strains substantially altered the community structure of the cigar wrapper. In particular, the abundance of dominant *Staphylococcus nepalensis* significantly increased under addition of strain DS101 and DS309 except for strain DS1 as dominant species. In addition, non-targeted metabolomics analyses showed that the exogenous microbial addition treatments demonstrated substantial variations in a variety of metabolites, including a reduced expression in the pathways related to the manufacture of polyphenols and unsaturated fatty acids (*p* ≤ 0.05). In conclusion, addition of bacteria DS1, DS101, and DS309 enhanced oxidation of polyphenols to brown quinones and oxidation of unsaturated fatty acids, resulting in membrane lipid peroxidation and then deepened the color of cigar wrappers. It provides a novel strategy to improve the color of cigar wrappers by utilizing additional bacteria in cigar fermentation.

**Supplementary Information:**

The online version contains supplementary material available at 10.1186/s40643-025-00921-5.

## Introduction

Cigars are a unique kind of tobacco product with a strong flavor and rich aroma (Geng et al. 2023). They make up around 2% of all tobacco products worldwide and cigar consumption is currently growing quickly in China (Liu et al. 2024). A completed cigar includes filler, binder, and wrapper (Li et al. 2015). The core of a cigar, known as the filler, is composed of several layers of tobacco leaves in precise ratios, which have an immediate impact on the cigar’s burnability, flavor, and aroma (Chen et al. 2019; Hu et al. 2022; Yao et al. 2022.). The binder keeps the filler leaves tightly packed and properly shaped. The wrapper protects the filler, and affects the cigar’s look and flavor. As the exterior of the cigar, the wrapper has an impact on the cigar’s quality, appearance and smoking flavor by its color and texture (Wang. 2015). Previous studies have demonstrated that an accelerating transformation process of polyphenols resulted in the deepening of the color of cigar wrappers (Guo et al. 2022). These fermentative metabolites improve the cigar’s flavor and quality (Zheng et al. 2022a). Consequently, the color of wrappers is typically closely linked to the fermentation process, and served as a significant indicator of the quality (Banožić et al. 2020).

It’s well known that numerous microbial species were identified in tobacco leaves, playing an essential role in the fermentation process of CTLs (Joshi et al. 2024). These microorganisms not only secrete a range of extracellular hydrolytic enzymes that speed up the breakdown of proteins, starch, cellulose, and other macromolecular components, and reduce the content of dangerous substances such as nicotine, but also boost the amount of aroma components such as dihydronediolactone and meglomaniacal trienolone to improve the quality of tobacco leaves (Xue et al. 2023; Wang et al. 2024;). Moreover, microorganisms strongly link to the color shift during fermentation of CTLs. For example, the amount of chlorophyll and polyphenols in tobacco leaves steadily declines during cigar fermentation, resulting in deepening the color of CTLs gradually (Chattopadhyay et al. 2021; Liu et al. 2022). Recently, fermentation quality of CTLs can be further improved by exogenously adding functional microorganisms relying on their function of producing enzymes and flavors (Jia et al. 2023.), displaying a wide range of potential applications (Liu et al. 2015). For instance, additional *Cyberlindnera fabianii* from CTLs decreased the overall alkaloid content while increasing the concentration of flavor compounds (Guo et al. 2024), and exogenous microorganisms (*Acinetobacter* sp. 1H8 and *Acinetobacter indicus* 3B2) improved the quality and flavor of cigar tobacco through metabolic role and affecting the microbial community structure and function (Zheng et al. 2022b). Although microbial fermentation research in CTLs has advanced significantly, especially on how microorganisms increase the production of aroma substances (Zheng et al. 2022b; Guo et al. 2024; Pei et al. 2025), relatively little is known about how microorganisms regulate the color of wrapper since the most studies have focused on the physical methods of controlling wrapper color (Zheng et al. 2022b; Yang. 2023).

The aim of this study was to explore the microbial resources and correlation of fermentative indices that deepen the color of cigar wrappers. Bacterial strains were isolated and screened from healthy CTLs, then three strains were used in stack fermentation of CTLs to investigate the influence of additional bacteria on the color change of cigar wrapper. A colorimeter was used to determine the color at the end of fermentation, and the correlation analysis was performed among color parameters, enzyme activities, microbial communities, and metabolites. This research displays a promising prospect to enhance wrapper color through application of additional microorganisms in cigar production.

## Materials and methods

### Experimental material and sites

The tobacco variety used in this study is ChuXue-80 (CX-80) from Enshi, Hubei province, China. The harvest, drying, and fermentation processes of CTLs were in accordance with the ‘Cigar Tobacco Leaf Production Technical Regulations’ of Enshi Branch, Hubei Tobacco Company, China. CTLs stack fermentation was performed in the Cigar Tobacco Fermentation Plant in Laifeng County, Hubei Province during 2023–2024.

### Color determination of CTLs

The color of fermented CTLs was determined by using a Leica precision colorimeter (SC-10, Shenzhen Sansi Technology Co., Ltd., China). Each leaf was systematically sectioned into upper, middle, and lower sections, with six symmetrically-distributed detection points positioned equidistantly along both sides of the midrib (serving as the axis of symmetry). Triplicate measurements of the CIELAB color space parameters—brightness value L*, redness value a*, and yellowness value b*—were recorded at each point, and the average of all readings was used as the chromaticity value for each sample (Papadopoulos et al. 2010).

1 $${\Delta}\text{E}\:=\:({\Delta}\text{L}^{*2}\:+{\Delta}\:\text{a}^{*2}\:+\:{\Delta}\text{b}^{*2})^{1/2}$$

The color of CTLs is indicated by the value of E*, and a darker color is indicated by a larger value of ΔE. The color change is visible by naked eyes when ΔE is more than 1.5 (Mara et al. 2023).

### Isolation of microorganisms from CTLs

To obtain the bacterial suspension, 5.0 g of CTLs was weighed at the drying and fermentation stages, mixed with 95 mL of sterile 0.85% saline water for grinding at 28 °C with shaking at 150 rpm for 24 h. After that, 10-fold series of dilutions were performed. Colonies were obtained from solid culture mediums, including R2A and YPDA at 28 °C. Various strains were purified and identified preliminarily based on 16S rRNA gene sequences after duplicate isolates were removed (Huang et al. 2020). All preliminarily identified isolates were stored at -80 °C for next use (Bhattacharjee et al. 2021).

### Screening and identification of microorganisms that modify color of CTLs after semi-leaf fermentation

Uniformly colored CTLs were selected and divided into two sections with the main vein as the center. For one section of the CTLs, a bacterial suspension was evenly sprayed onto their surfaces at a bacterial inoculum level of 1 × 10⁷ CFU/g of CTLs, whereas for the other section, an equivalent amount of water was sprayed onto their surfaces to serve as a control (Song et al. 2024). Each treatment was performed as five replicates. Subsequently, the piles were placed in the fermentation room under the conditions of 24.6 °C and 97.4% relative humidity, fermented for 30 days. Automated temperature and humidity monitoring devices were positioned at various locations within the stacks. The fermentation process was considered completed when the temperature within the stacks stabilized (Li et al. 2020).

After the ΔE value of each cigar wrapper sample was measured, the three added strains in the treatment groups with a ΔE value greater than 1.5 were selected for subsequent experiments. To investigate the morphological characteristics of the three added strains, a 1% inoculum of each was cultured in 5 mL LB medium at 28 °C and 150 rpm for 24 h. Subsequently, 100 µL of the 10⁻¹⁰ dilution of each culture was spread onto LB Solid Medium. Concurrently, 2 mL of the bacterial culture was sampled and centrifuged at 12,000 rpm for 5 min at room temperature to collect the cells. Concurrently, 2 mL of the bacterial culture was sampled, and the cells were prepared for scanning electron microscope according to the method described by Zhou (Zhou et al. 2025). Finally, the dehydrated cells were dried using a vacuum freeze-dryer (Ningbo Yinzhou Sjia Lab Equipment Co., Ltd., China) and visualized under a scanning electron microscope (SEM, JEOL Model JSM-6390LV, Tokyo, Japan).

The taxonomic categories of screened strains were identified based on the genomic sequences as previously described method (Wang et al. 2019a). The average nucleotide identity (ANI) values were computed by means of the EzBioCloud website (www.ezbiocloud.net/tools/ani). The digital DNA-DNA hybridization (dDDH) values were determined using the Genome-to-Genome Distance Calculator (version 3.0) webserver with the recommended Formula 2 (http://ggdc.dsmz.de/ggdc.php/). The phylogenetic tree based on the core genes of the genome was constructed in accordance with the previously described method (Wang et al. 2019a).

### Exogenous microorganism application in large stack fermentation of cigar wrappers

In each experimental group, a total of fifty intact cigar wrappers were uniformly sprayed with a specific bacterial solution at an inoculum concentration of 1 × 10⁷ CFU/g. In parallel, the control group was sprayed with an equivalent volume of water. Each group was performed as three replicates. Subsequently, all the cigar wrappers were placed into stacks with a mass of 2000 kg for upscaled fermentation. Whenever the daily recorded temperature within the stacks reached 50 °C to 55 °C, the stacks were turned over following the standard protocol of ‘turning the top and bottom, turning the inside and outside’. The stack fermentation was maintained natural humidity of 60–80% (Li et al. 2019). Subsequently, they were re-stacked, and this operation was repeated within the range of three to five times (Li et al. 2020). Approximately 90 days after fermentation, the temperature within the stacks remained substantially stable, it was considered as the end of fermentation process of the CTLs. Finally, the color parameters of each group of cigar wrappers were accurately determined and the total color difference value (ΔE) was calculated as described in Sect. ‘Color determination of CTLs’.

### Determination of physiological and biochemical indices for cigar wrappers after large stack fermentation

UV spectrophotometry (UV1900, Shanghai AOE Instruments Co., Ltd., China) was adopted to precisely determine the content of plastid pigment within the cigar wrappers of each group (Wu et al. 2020). Concurrently, the titrimetric method was employed to accurately measure the activities of polyphenol oxidase (PPO) and ascorbic acid oxidase (AAO) (Sallam et al. 2021). Subsequently, a comprehensive examination including oil content, thickness, and completeness was conducted on the difference between the cigar wrappers of each treatment and those of the control (Zhao et al. 2023a).

### Analysis of bacterial communities in cigar wrappers after large stack fermentation under additional bacteria

For each treatment, precisely 1 g of fermented cigar wrappers with stems removed was meticulously collected (Chen et al. 2018a). Subsequently, the collected sample was subjected to grinding in liquid nitrogen. Thereafter, the ground samples were carefully placed into separate sterile bags, maintained on ice, and subjected to amplicon sequencing without delay (Karakas et al. 2023). The full length of 16S rRNA gene amplicon mixtures of each sample were employed for the construction of sequencing libraries by utilizing the Pacific Biosciences SMRTbell™ Template Prep kit 1.0, with the primers used being 27F (3’-AGAGTTTGATCMTGGCTCAG) and 1492R (5’-ACCTTGTTACGACTT) (Yang et al. 2018). Subsequently, the resultant sequencing libraries were sequenced on a PacBio Sequel II platform. All amplicon sequencing operations were conducted by Shanghai Sangyo Bio. The PacBio raw sequence reads were processed through the application of SMRT Link Analysis software version V9 to obtain circular coherence sequences. Specifically, the minimum number of passes was configured to be 3, and the minimum prediction accuracy was stipulated to reach 99%. OTUs were clustered in accordance with a similarity threshold of 98.65% by means of UPARSE, and chimeric sequences were identified and removed with the assistance of UCHIME. The UCLUST algorithm, with a confidence threshold configured at 0.8, was adopted to classify the OTU representative sequences. Leveraging this classification, the bacterial community composition of each sample was meticulously analyzed through a comparison with the Silva 16S rRNA database.

### Analysis of metabolomic profiles in cigar wrappers after large stack fermentation under additional bacteria

100 mg of fermented cigar wrappers were first ground in liquid nitrogen and then carefully transferred into a centrifuge tube. After precisely 500 µL of 80% methanol aqueous solution was introduced, the mixture was subjected to vortexing and shaking operations. Subsequently, it was performed in an ice bath for 5 min, and centrifuged at 15,000 g for 20 min under 4 °C. Then, a specific volume of the supernatant was added to mass spectrometry-grade water to achieve a methanol concentration of 53%, and centrifugation was performed at 15,000 g for 20 min under 4 °C. Finally, the supernatant was collected and then carefully injected for LC-MS/MS analysis. The raw data obtained from mass spectrometry were processed using the Compound Discoverer 3.1 data processing software, with the aim of obtaining the mass-to-charge ratio (m/z), retention time, and peak area of the substances. The identification of metabolites was carried out through a comparative analysis with the mzCloud, mzVault, and Masslist databases. Subsequently, the identified metabolites were subjected to functional and taxonomic annotation by means of the Kyoto Encyclopedia of Genes and Genomes (KEGG), Human Metabolome Database (HMDB, https://hmdb.ca/metabolites/), and LIPID MAPS database, thereby facilitating a comprehensive understanding of their biological characteristics (Kanehisa et al. 2017; O’Donnell et al. 2019; Wishart et al. 2022).

### Data analysis

One-way analysis of variance (ANOVA) was performed to conduct a comprehensive analysis of the color parameters, enzyme activity, as well as the plastid pigment content by using SPSS software (version 25.0.0.2). Subsequently, the results were visualized via GraphPad Prism 8 (version 8.2.1). Redundancy analysis (RDA), a multivariate direct gradient analysis method, was calculated by Canoco (version 4.5) to elucidate the relationships among microorganisms, enzyme activities, metabolites, and total color difference.

SIMCA software (version 14.0) was employed to rigorously examine the significant differences among the metabolites of each group of cigar wrappers. Specifically, Principal Component Analysis (PCA) and Partial Least Squares Discrimination Analysis (PLS-DA) were executed in succession. Subsequently, taking into account the contribution of each group, the differential abundance metabolites were precisely screened out by adhering to the Variable Importance in the Projection (VIP) and considering the significance of inter-group variation. Ultimately, a hypergeometric test was applied to the metabolites for the purpose of conducting KEGG pathway enrichment analysis. Subsequently, the resultant metabolic pathway enrichment results were retrieved and further subjected to in-depth analysis using Origin 2024.

## Results

### Identification of microorganisms that improve the color of cigar wrappers

**Fig. 1:**
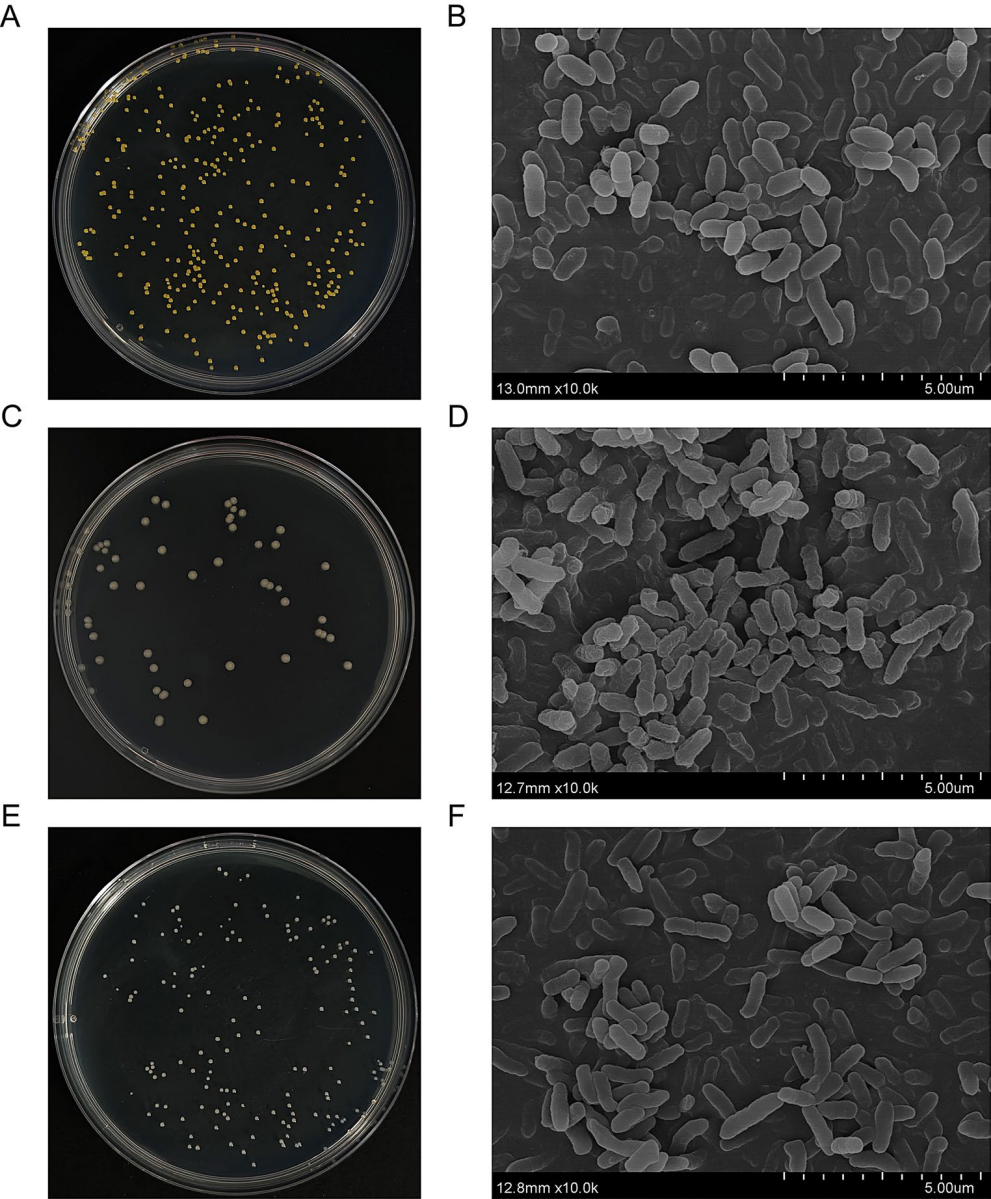
Morphological trait of strains DS1, DS101, and DS309. (**A**, **C**, **E**) Colonies of strains DS1, DS101, and DS309 on LB plate. (**B**, **D**, **F**) Ultrastructural observations of strains DS1, DS101, and DS309 by Scanning Electron Microscopy

The half-leaf fermentation showed that the color of the front or back side of cigar wrappers was significantly enhanced added with three strains DS1, DS101, and DS309 in comparison to that of the control (Fig. S1). Specifically, this increment surpassed 1.5, thereby indicating visible color alterations by the naked eyes. Consequently, the whole genome of those three strains was sequenced for identification of their taxonomic status. In addition, their morphological traits were observed (Fig. 1), in particular, SEM images showed they all are rod - shaped cells.

**Fig. 2:**
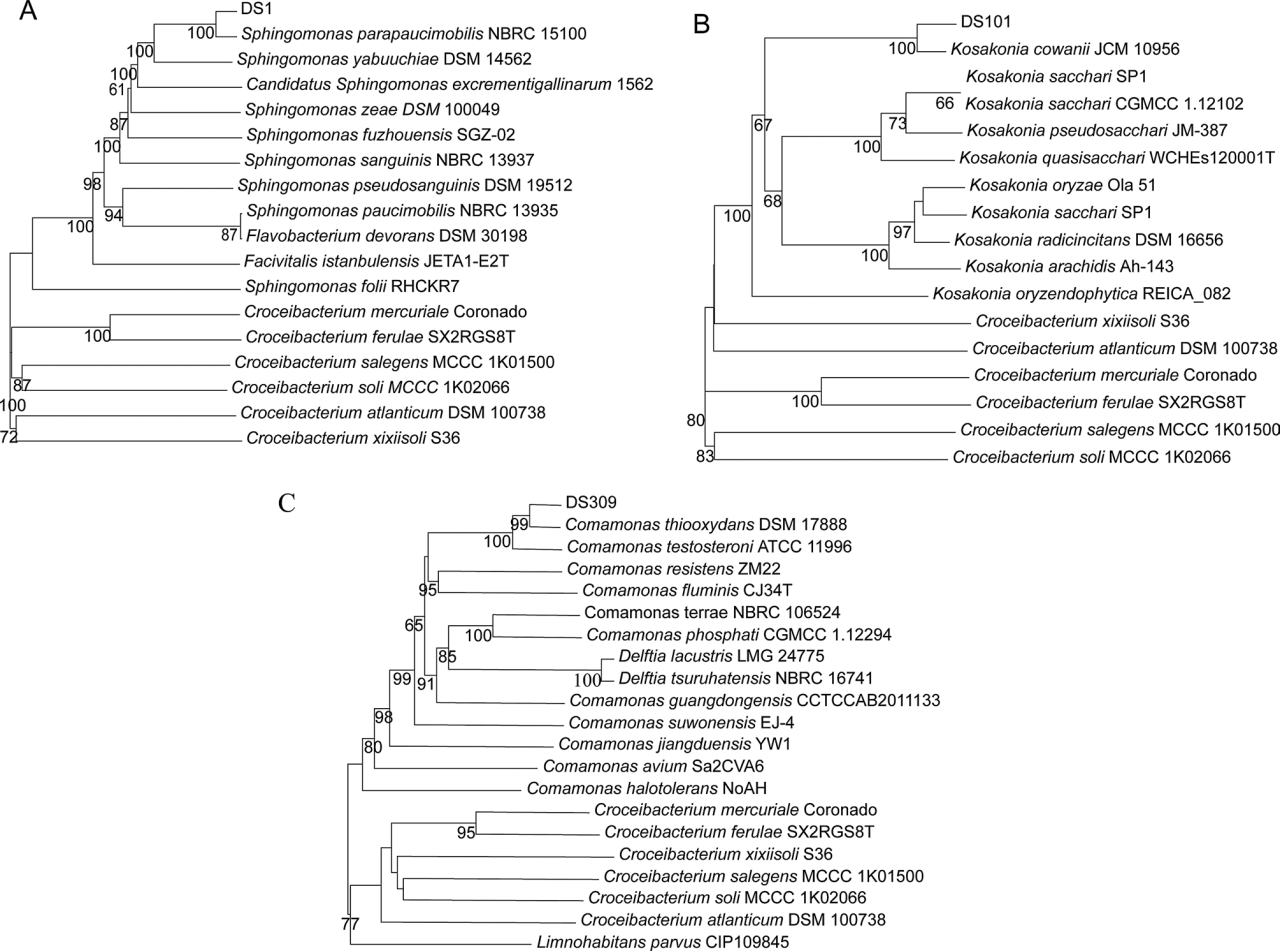
Maximum- likelihood phylogenomic trees based on genomic sequences. (**A**) Genomic phylogenetic tree of strain DS1, (**B**) Genomic phylogenetic tree of strain DS101, (**C**) Genomic phylogenetic tree of strain DS309. DS1, *Sphingomonas parapaucimobilis*; DS101, *Kosakonia cowanii*; DS309, *Comamonas thiooxydans*

On the whole-genome phylogenetic tree, strain DS1 was clustered in proximity to *Sphingomonas parapaucimobilis* (Fig. 2A). In accordance with the species thresholds of 95% average nucleotide identity (ANI) and 70% digital DNA-DNA hybridization (dDDH), strain DS1 was identified as *Sphingomonas parapaucimobilis* as ANI values of 97.63% and dDDH values of 76.2%, respectively (Tables S1). Likewise, strain DS101 was identified as *Kosakonia cowanii* as 96.89% of ANI value and 72.2% of dDDH value (Fig. 2B, Tables S2). Ultimately, strain DS309 was identified as *Comamonas thiooxydans* (Fig. 2C, Table S3).

### Addition of exogenous microorganisms deepen the color of cigar wrappers under large stack fermentation

**Fig. 3:**
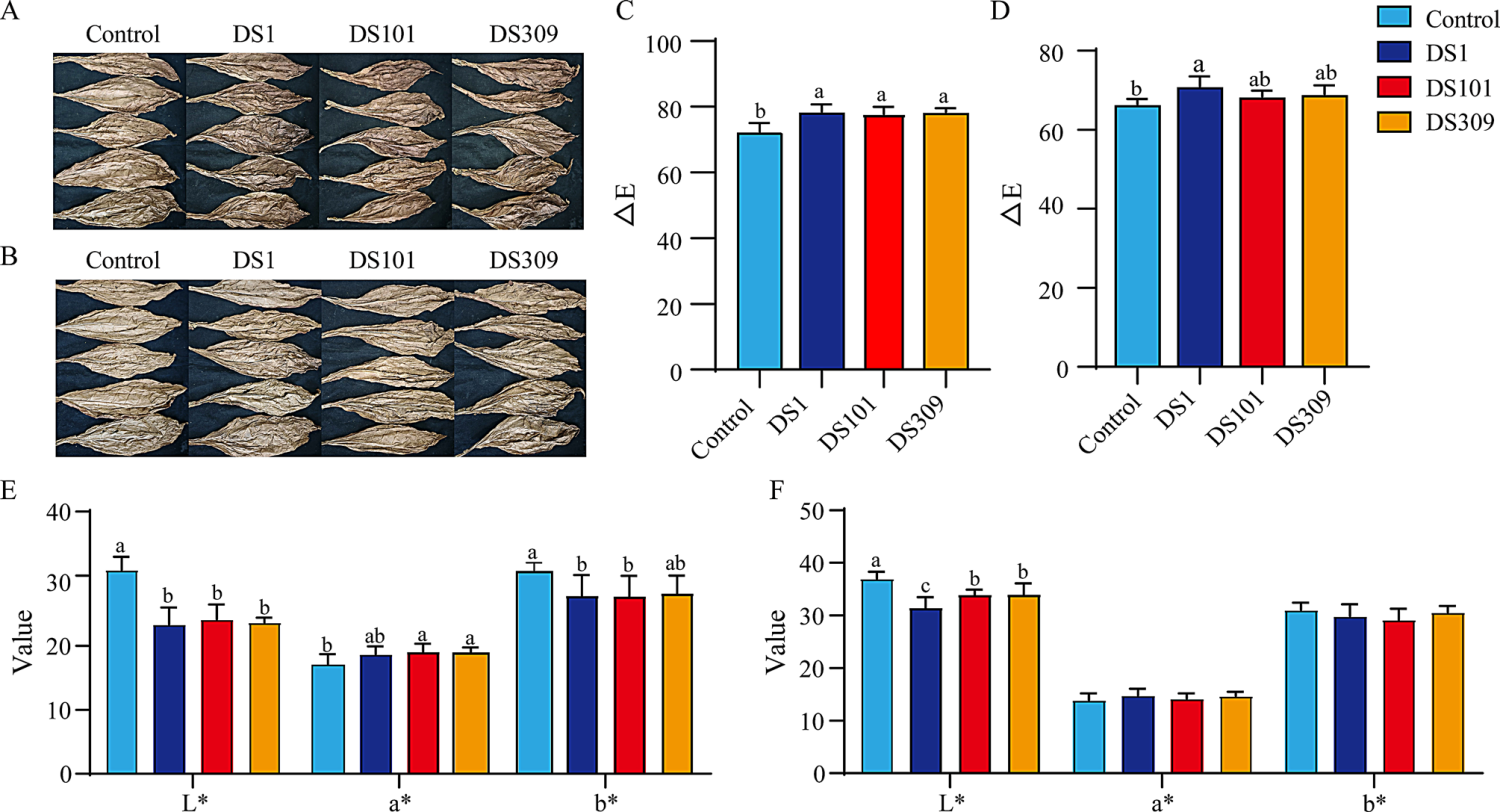
The color shift of front and back side of cigar wrappers added with bacteria under large stack fermentation. DS1, *S. parapaucimobilis*; DS101, *K. cowanii*; DS309, *C. thiooxydans*. (**A**) Front color image, (**B**) Back color image, (**C**) The total color difference value of front side, (**D**) The total color difference value of back side, (**E**) Front chromaticity value, (**F**) Back chromaticity value. Different letters indicate significant differences (*p* ≤ 0.05)

The chromaticity values and discoloration index of the front side of cigar wrappers after large stack fermentation were significantly elevated under the addition of the three strains in comparison to the control. These color alterations were clearly discernible to the naked eyes because the increase of the total color difference values (∆Es) were greater than 1.5 (Fig. 3A and B), i.e., the increments of ∆E were 6.02, 5.35 and 5.92 under strain *S. parapaucimobilis*m DS1, *K. cowanii* DS101 and *C. thiooxydans* DS309, respectively (*p* ≤ 0.05) (Fig. 3C). In detail, the brightness value L* was significantly lower in the three treatments than that in the control. The redness value a* was significantly higher in the *K. cowanii* DS101 and *C. thiooxydans* DS309 treatments relative to the control. Conversely, the yellowness value b* was significantly lower in the *S. parapaucimobilis*m DS1 and *K. cowanii* DS101 treatments than that in the control (*p* ≤ 0.05) (Fig. 3E). Likewise, ∆E values of the back side were 4.51, 1.89, and 2.52 under addition of strain *S. parapaucimobilis*m DS1, *K. cowanii* DS101 and *C. thiooxydans* DS309, respectively (Fig. 3D). The brightness value L* of all treatments was significantly lower than that of the control, whereas the redness value a* and yellowness value b* did not show significant differences (Fig. 3F). Moreover, there were no disparities in physical aspects such as leaf identity, oil content, and completeness between the treatments added with those three strains and the control after stack fermentation (Table S4). Collectively, the addition of these three strains led to a reduction in the brightness values and an augmentation of the total color difference values, consequently deepening the color of cigar wrappers.

### Addition of exogenous microorganisms alter the activities of key enzymes in cigar wrappers under large stack fermentation

**Fig. 4:**
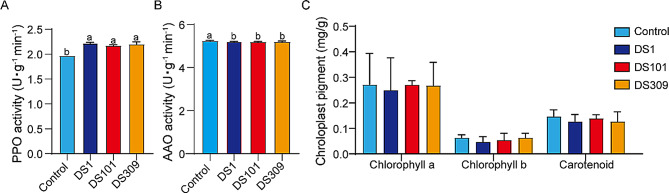
The content of essential enzyme activities and plastid pigment in cigar wrappers treated with different microorganisms under large stack fermentation. DS1, *S. parapaucimobilis*; DS101, *K. cowanii*; DS309, *C. thiooxydans*. (**A**) Polyphenol oxidase (PPO) activity, (**B**) Ascorbic acid oxidase (AAO) activity, (**C**) Plastid pigment content. Different letters indicate significant differences (*p* ≤ 0.05)

The activities of essential enzymes related to color change were determined after the end of large stack fermentation of cigar wrappers. The polyphenol oxidase (PPO) activity was significantly increased under respectively additional three strains compared to the control (*p* ≤ 0.05) (Fig. 4A). In contrast, the ascorbic acid oxidase (AAO) activities added with three additional bacterial strains were significantly lower than that of the control (*p* ≤ 0.05) (Fig. 4B). However, the contents of the plastidic pigments including chlorophyll a, chlorophyll b, and carotenoid didn’t display differences between the exogenous bacterial treatments and the control (Fig. 4C).

### Additional bacteria alter the bacterial community structure in cigar wrappers under large stack fermentation

**Fig. 5:**
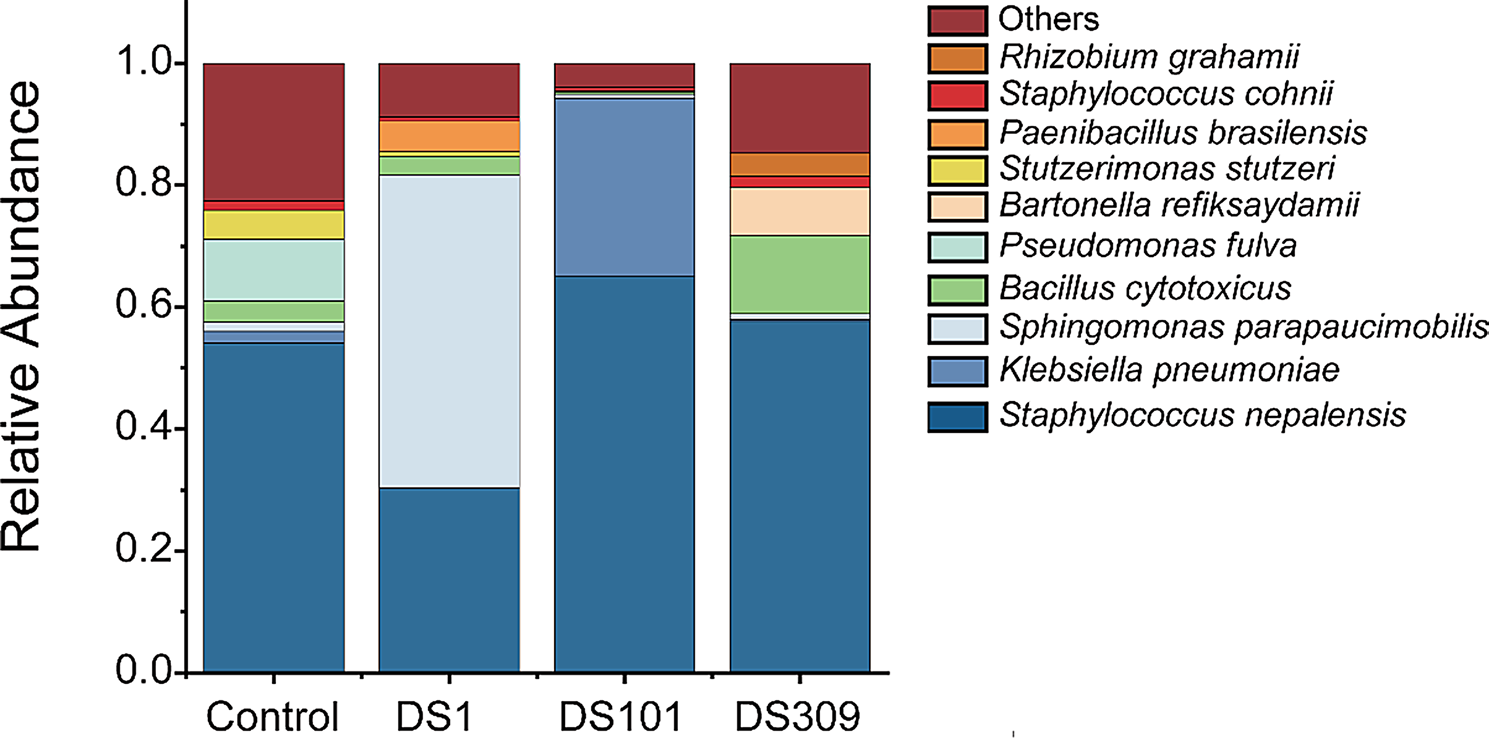
Bacterial species composition based on the full length of 16S rRNA gene sequences in cigar wrappers with different microbial treatments after large stack fermentation. DS1, *S. parapaucimobilis*; DS101, *K. cowanii*; DS309, *C. thiooxydans*

Based on the full length of 16S rRNA gene sequences, significant differences were discerned in the composition of dominant species among all cigar wrapper samples subjected to various treatments (Fig. 5). *Staphylococcus nepalensis* was manifested as the dominant taxon across all samples. Nevertheless, its abundance was solely down-regulated under additional *S. parapaucimobilis* DS1, whereas the abundance of *S. nepalensis* was slightly induced by addition of the other two strains in comparison with the control. It’s interesting that the supplementation of strain *S. parapaucimobilis* DS1 led to the replacement of *S. nepalensis* as the dominant taxon. Moreover, both *Bacillus cytotoxicus* and *Bartonella refiksaydamii* showed a substantial elevation in abundance within the treatment with the addition of *C. thiooxydans* DS309. By contrast, the two species retained a relatively low abundance level in the control as well as in the treatments with the addition of strain *S. parapaucimobilis* DS1 and *K. cowanii* DS101. It is noteworthy that the abundances of *Pseudomonas fulva* and *Stutzerimonas stutzeri* were both reduced in the cigar wrappers added with those three exogenous microorganisms.

### Additional bacteria change metabolite composition and metabolic pathways in cigar wrappers after large stack fermentation

**Fig. 6:**
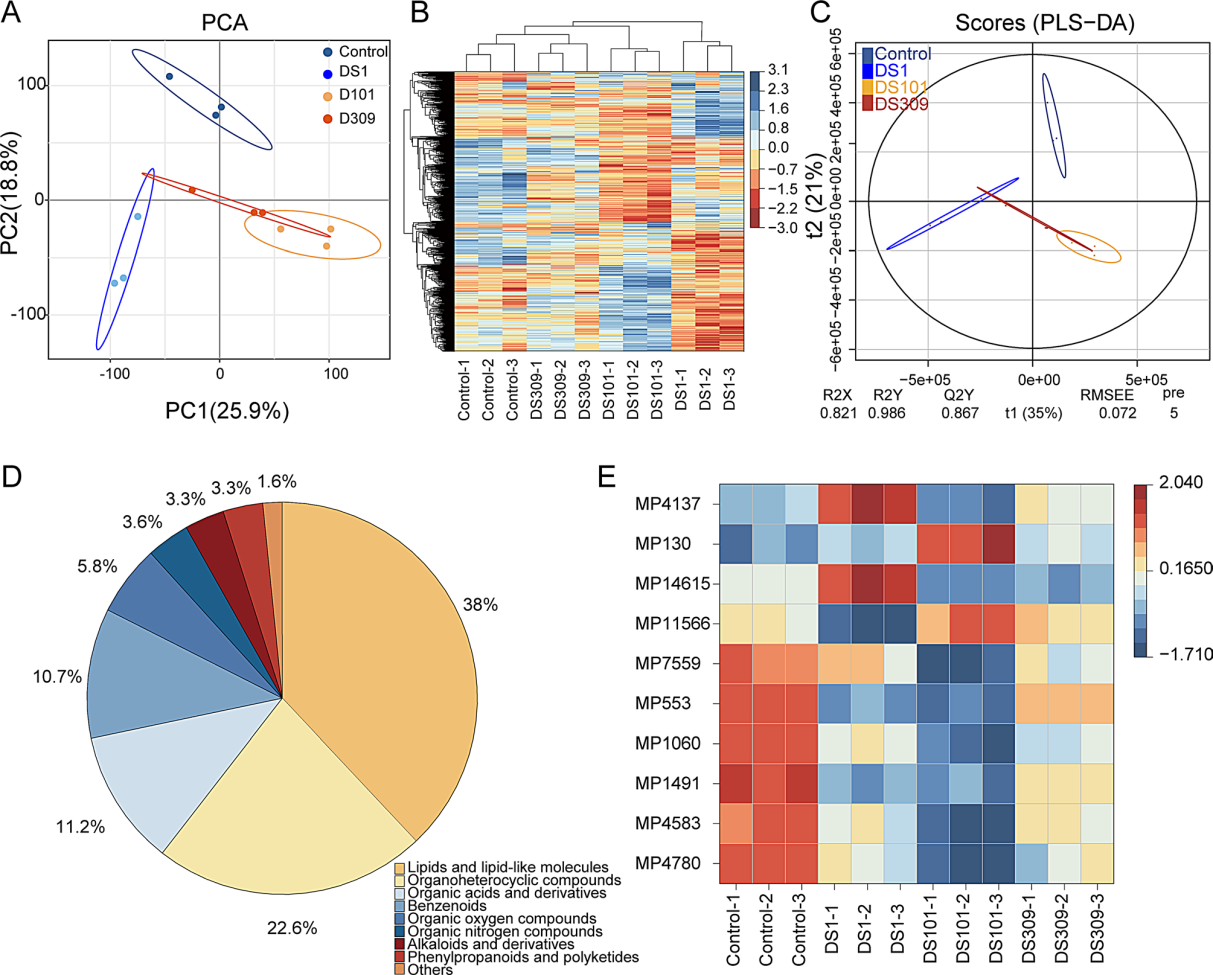
Full analysis of metabolites and metabolic profiles in cigar wrappers with different microbial treatments after large stack fermentation. DS1, *S. parapaucimobilis*; DS101, *K. cowanii*; DS309, *C. thiooxydans*. (**A**) Principal component analysis (PCA). (**B**) Cluster analysis. (**C**) Partial Least Squares Discrimination Analysis (PLS-DA). (**D**) Metabolite share in HMDB chemical classification. (**E**) Heatmap of top 10 differential abundance metabolite clustering among groups of tobacco leaves. The top 10 were selected from differential metabolites (*P* < 0.05; VIP > 1) based on importance scores from a random forest model. MP4137: 6, 9R,10R-trihydroxy-7E12Z,15Z-octadecatrienoic acid. MP130: L-Isoleucine. MP14615: (6 S)-16-hydroxy-6-(2-hydroxypropan-2-yl)-7, 11, 19-trioxapentacyclo [10.8.0.02,10.04,8.013,18] icosa-1(12), 2(10), 3,8,13(18), 14,16-heptaen-20-one. MP11566: 4-Propyl-N-(2-pyridinylmethyl) benzenesulfonamide. MP7559: 7-(2-hydroxypropan-2-yl)-1, 4a-dimethyl-1, 2, 3, 4, 4a, 9,10,10a-octahydrophenanthrene-1-carboxylic acid. MP553: Lycoverticine. MP1060: 15-Hexadecynoic acid. MP1491: 16-hydroxyfurazabol. MP4583: Trans-Anethole. MP4780: Methyl myristoleate

Cluster analysis and Principal Component Analysis (PCA) were performed on the four groups of cigar wrapper samples (Fig. 6A and B). The results demonstrated that the three biological replicates within each treatment were grouped into one unit, indicating good intra-sample reproducibility and more distinct inter-sample differences. To avoid the interference of random sampling and external factors on the classification information, Partial Least Squares Discrimination Analysis (PLS-DA) was also implemented in this study (Fig. 6C). The results revealed that the samples of the control were distributed in the upper half of the confidence interval, while the samples of the treatments were distributed in the lower half of the confidence interval. This indicated that the PLS-DA model could effectively distinguish between the cigar wrapper samples of each group, and there was a substantial difference in the metabolites between the treatments and the control. In summary, the above four cigar wrapper treatments featured high sample quality and reliable metabolome data, which were suitable for subsequent analyses.

Upon combining the positive and negative ion models, among the metabolites identified in the cigar wrapper samples, lipid and lipid-like molecules such as linoleic acid and linolenic acid accounted for 38.0%; organic heterocyclic compounds accounted for 22.6%; organic oxides accounted for 5.8%; organic nitrogen compounds accounted for 3.6%; benzene-type compounds accounted for 10.7%; phenylpropanes and polyketides accounted for 3.3%; nucleosides, nucleotides and their analogues accounted for 2.0%; alkaloids and their derivatives accounted for 3.3%; and others accounted for 0.7% (Fig. 6D). Moreover, exogenous microbial treatment altered the tobacco metabolic profile. After screening for differential metabolites (VIP > 1, *P* < 0.05), the top 10 prioritized by random forest importance were plotted in Fig. 6E.

Metabolic pathway analysis was also conducted on the differential metabolites in cigar wrappers among different treatments. The top 20 pathways characterized by the smallest p - value, the ones most significantly enriched, were chosen for presentation. The KEGG pathways were classified into four principal categories, including ‘metabolism’, ‘environmental information processing’, ‘genetic information processing’, and ‘information processing’. Notably, ‘metabolism’ encompassed the largest number of differential metabolite pathways annotated within the exogenous microbial treatment group.

**Fig. 7:**
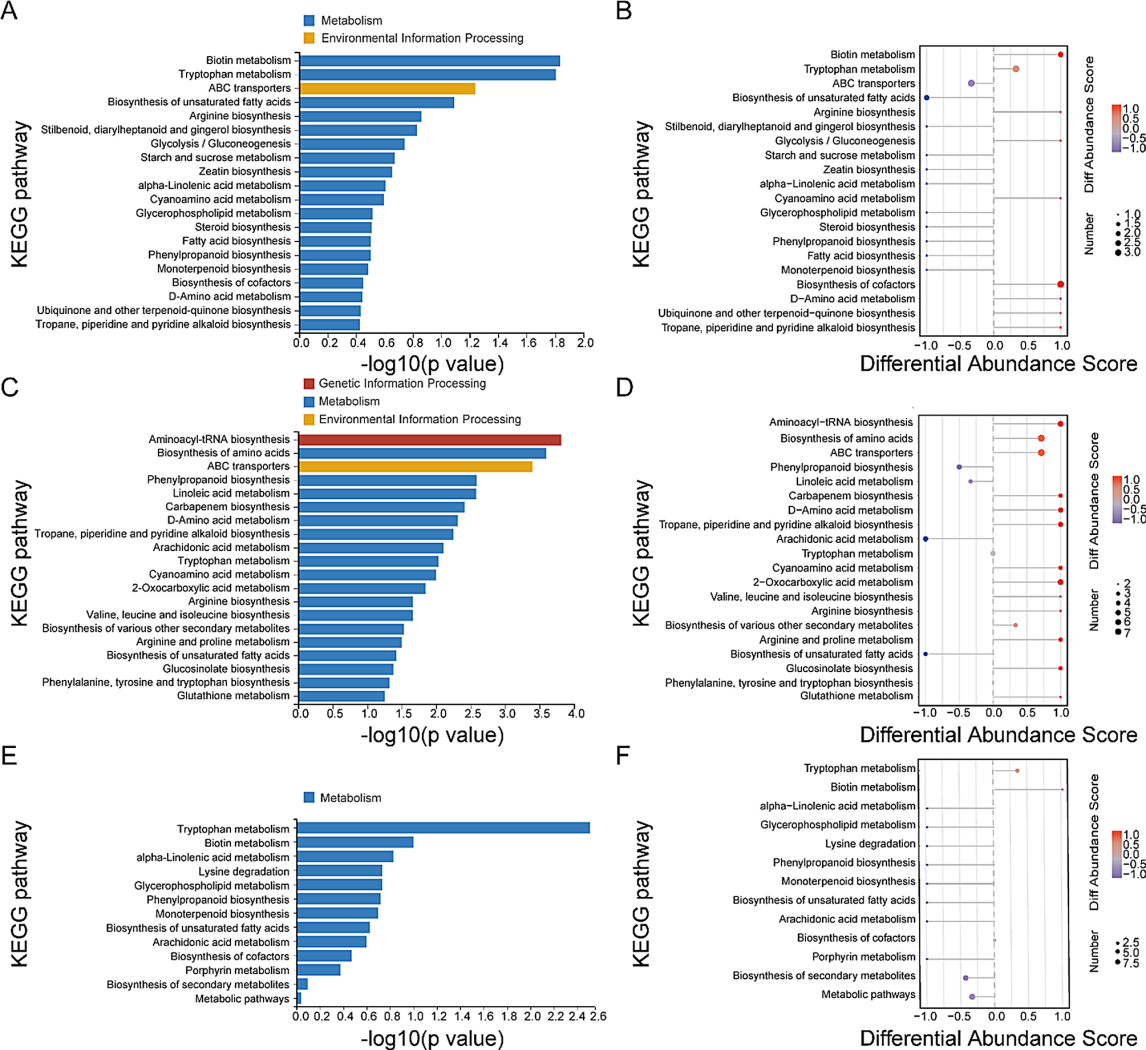
KEGG classification and enrichment of differential metabolites. (**A**, **C**, **E**) KEGG enrichment of differential metabolites between *S. parapaucimobilis*m DS1 and Control, *K. cowanii* DS101 and Control, and *C. thiooxydans* DS309 and Control. (**B**, **D**, **F**) KEGG enrichment differential abundance analysis of differential metabolites between *S. parapaucimobilis*m DS1 and Control, *K. cowanii* DS101 and Control, and *C. thiooxydans* DS309 and Control

The KEGG enrichment results of differential metabolites showed the top 10 pathways with the smallest Q-values within the *S. parapaucimobilis*m DS1 and *K. cowanii* DS101 treatments, and the top 13 pathways in the *C. thiooxydans* DS309 treatments (Fig. 7A, C, and E). Regarding lipid metabolism, the biosynthesis of unsaturated fatty acids was observed to be down-regulation under the groups added with the three microorganisms. That is to say, the number of down-regulated differential metabolites as ‘Biosynthesis of unsaturated fatty acids’ were one, three, and one under *S. parapaucimobilis*m DS1, *K. cowanii* DS101 and *C. thiooxydans* DS309 treatment, respectively. In contrast, there is only a metabolite as ‘α - Linolenic acid metabolism’ showed down regulation under *S. parapaucimobilis*m DS1 and *C. thiooxydans* DS309 treatments. Additionally, multiple metabolic pathways demonstrated accelerated polyphenol oxidation pathways within the exogenously added microbial treatments. The pathways of ‘Phenylpropanoid biosynthesis’ exhibited a down-regulation under all exogenous microbial treatments. Conversely, the pathway of ‘Ubiquinone and other terpenoid - quinone biosynthesis’ showed an up-regulation under *S. parapaucimobilis*m DS1 treatment (Fig. 7A, C, and E).

### Correlations among microorganisms, metabolites, enzyme activities and total color difference in cigar wrappers of different treatments

**Fig. 8:**
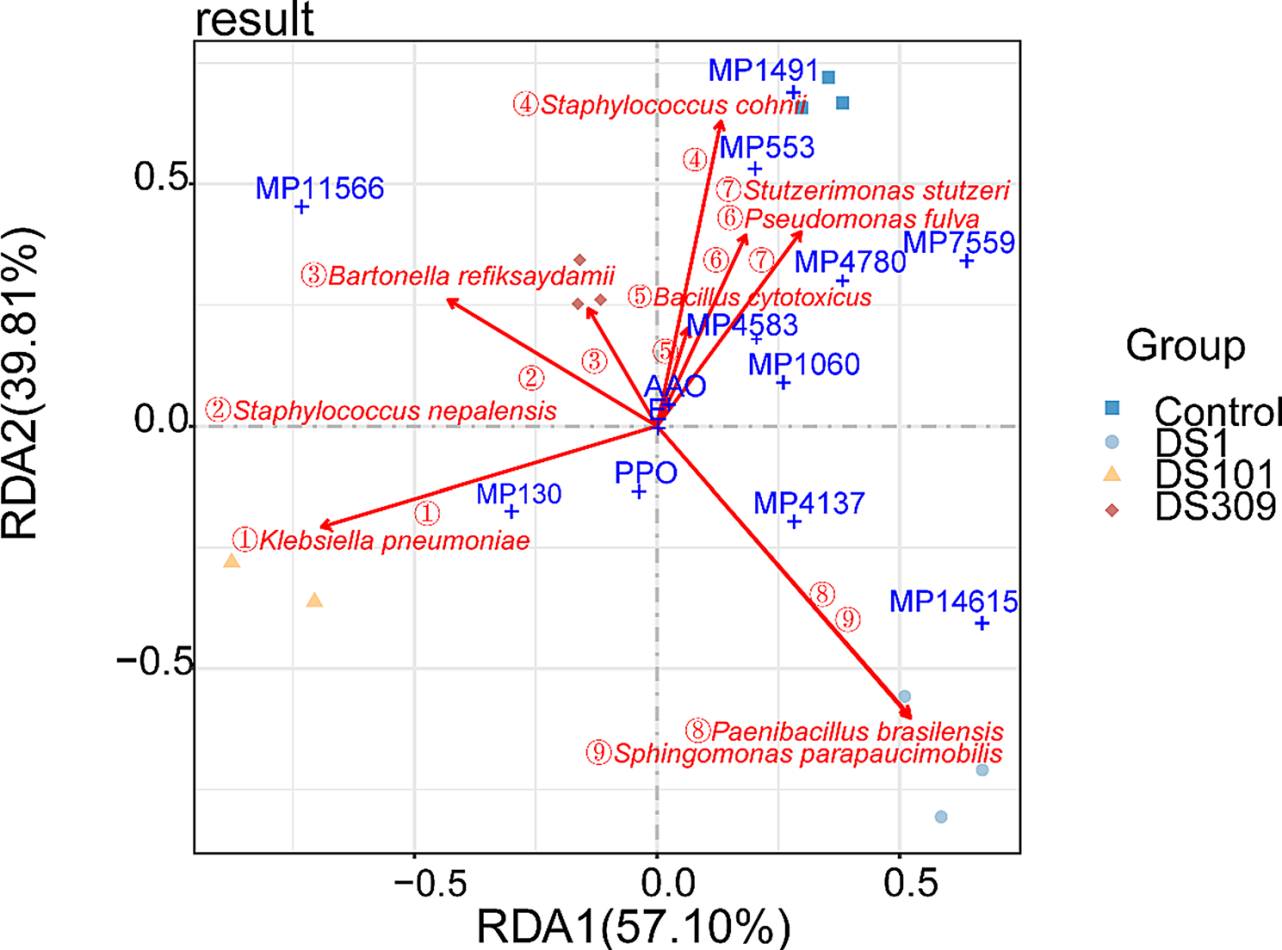
Redundancy analysis between microorganisms, metabolites, enzyme activities and total color difference in different microbial treatments of cigar wrappers. DS1, *Sphingomonas parapaucimobilis*; DS101, *Kosakonia cowanii*; DS309, *Comamonas thiooxydans*. MP4137: 6, 9R, 10R-trihydroxy-7E12Z,15Z-octadecatrienoic acid. MP130: L-Isoleucine. MP14615: (6 S)-16-hydroxy-6-(2-hydroxypropan-2-yl)-7, 11, 19-trioxapentacyclo [10.8.0.0^2^,1^0^.0^4^,^8^.0^13^,^18^] icosa-1(12), 2(10), 3, 8, 13(18), 14, 16-heptaen-20-one. MP11566: 4-Propyl-N-(2-pyridinylmethyl) benzenesulfonamide. MP7559: 7-(2-hydroxypropan-2-yl)-1, 4a-dimethyl-1, 2, 3, 4, 4a, 9, 10, 10a-octahydrophenanthrene-1-carboxylic acid. MP553: Lycoverticine. MP1060: 15-Hexadecynoic acid. MP1491: 16-hydroxyfurazabol. MP4583: trans-Anethole. MP4780: Methyl myristoleate. PPO: polyphenol oxidase. AAO: ascorbic acid oxidase

Redundancy analysis (RDA) was employed to explore the correlations among microorganisms, metabolites, enzyme activities, and total color difference values. The results showed that all groups were distributed across distinct regions, indicating significant differences in microbial communities, metabolites, total color difference values, as well as enzyme activities among these groups (Fig. 8). Under the *S. parapaucimobilis*m DS1 treatment, *S. parapaucimobilis* as the dominant strain displayed a negative correlation with the lipid compound MP1491, a positive correlation with the polycyclic compound MP14615, and a robust correlation with PPO. Under the *C. thiooxydans* DS309 treatment, *B. cytotoxicus* as a relatively higher abundance showed a pronounced correlation with AAO, displaying a significant impact on the oxidation reactions of polyphenolic compounds during the tobacco fermentation process. *S. nepalensis* as abundant species in both the DS101 and DS309 groups, showed a positive correlation with the sulfonamide compound MP11566. By contrast, *P. fulva* and *S. stutzeri*, whose abundances were downregulated across all three groups, demonstrated a positive correlation with the alkaloid MP553 to degrade alkaloids served to alleviate tobacco irritation and enhance the smoking flavor.

## Discussion

Cigar wrapper color and uniformity are among the crucial indicators for the evaluation and grading of cigar tobacco quality (Xu et al. 2003). In contrast to roasted tobacco, the darkening of cigar color not only does not significantly impair the quality of tobacco but also enhances its exterior quality (Fan et al. 2023). Upon undergoing fermentation, the color shift of CTLs was considered as an outward indication of the transformation and decomposition processes of substances within the tobacco leaves (Zhang et al. 2024). As the fermentation progresses, the contents of chlorophyll and carotenoids experience a continuous decline, while enzymatic browning and Maillard reactions are instigated, thereby giving rise to the production of quinones and melanoidins that impart the brown color to the tobacco leaves (Ito et al. 2008). The variations in pigment content, in conjunction with enzymatic browning and Maillard reactions, constitute the principal factors contributing to the alteration in tobacco color, and these are closely linked with the functions of polyphenols and antioxidant enzymes (Pan et al. 2023). In this study, the exogenous addition of three distinct bacteria, namely *S. parapaucimobilis*m DS1, *K. cowanii* DS101, and *C. thiooxydans* DS309, induced a deepening of the color of cigar wrapper leaves during the fermentation process (Fig. 3C and D). Notably, quality indicators such as oil content, thickness, and leaf integrity remained unaltered (Table S4). This result provides a novel strategy regarding the artificial exogenous addition of microorganisms to facilitate the color alteration of cigar wrapper leaves during fermentation.

To unravel the causes underlying the color changes, an investigation was conducted into how the exogenous microorganisms influenced the bacterial flora, enzyme activities, and metabolites (Figs. 4 and 5). The exogenous addition of all three bacterial strains led to a significant elevation in the activity of polyphenol oxidase (PPO) in tobacco (Fig. 4A). Moreover, all three exogenously added microbial treatments of CTLs displayed down-regulation of the phenylpropane biosynthesis pathway (Fig. 7B, D, and F). It has been established that the phenylpropane metabolic pathway plays a principal role in the synthesis of polyphenolic compounds (Chen et al. 2018b; Wang et al. 2019b). As a consequence, the increase in PPO activity resulted in a diminution of the polyphenol content in CTLs. Moreover, an elevation in PPO activity exerts an influence on the color change of CTLs during fermentation by facilitating the breakdown of polyphenols through enzymatic browning processes, thereby giving rise to brown quinones (Singh et al. 2021). This color response was further corroborated, for instance, by the up-regulation of a quinone production pathway in the *S. parapaucimobilis*m DS1 treatment (Fig. 7B). This result is consistent with recent studies that have identified a correlation between phenolic metabolism and the intensity of browning during tobacco processing (Mao et al. 2021). Previous studies have also demonstrated that *Sphingomonas* sp. is capable of expressing polyphenol oxidases and degrading polyphenols such as chlorogenic acid (Wibowo et al. 2021), and that *Comamonas* sp. participates in phenolic oxidation processes during the breakdown of aromatic compounds, thereby manifesting a partial overlap of function with PPOs (Zhou 2007). These findings support the microbial mechanism of regulating tobacco color through the polyphenol oxidation pathway (Chen et al. 2018b; Wang et al. 2019b; Singh et al. 2021; Mao et al. 2021; Wibowo et al. 2021). In addition, exogenous microbial additions led to a decrease in AAO (ascorbic acid oxidase) activity (Fig. 4B) and an increase in oxidative stress. AAO is involved in the antioxidant defense system and serves to safeguard plant cells from oxidative damage by scavenging reactive oxygen species (Díaz-Vivancos et al. 2010). The oxidation of unsaturated fatty acids increased membrane lipid peroxidation, resulting in browning of plant leaves (Bai et al. 2023; Hong et al. 2023). This was confirmed by the result of metabolomic analysis that unsaturated fatty acids, such as linoleic acid and linolenic acid, were significantly decreased in the exogenously added microbial treatments of CTLs (Fig. 7B, D, and F). In particular, the lipid compound MP1491 (16 - hydroxyfurazabol) was significantly decreased under strain *S. parapaucimobilis*m DS1 treatment (Fig. 8), further indicating that the reduction in lipid content contributes to the deepening of the color of CTLs. Taken together, this study elucidated the mechanism that the increase of polyphenol oxidation to brown quinones and oxidation of unsaturated fatty acids cooperate to jointly regulate the color of CTLs upon the addition of exogenous strains.

Previous studies have reported that color variations of CTLs are closely correlated with the degradation of chlorophyll and carotenoids during CTLs fermentation (Liu et al. 2022; Zhao et al. 2023b; Meng et al. 2024). In this case, only a slight variance was observed in the amount of chlorophyll in CTLs added with exogenous strains compared to the control (Fig. 4C). This might be ascribed to the fact that the measurements in this study were taken at the end of the fermentation period, at which point there were no longer discrepancies between the treatments and the control. In fact, the degradation of carotenoids and chlorophyll predominantly occurs during the early fermentation stage, Chlorophyll a and b decrease rapidly within the first 0–10 days of fermentation, with slow and insignificant changes observed after day 25 (Liu et al. 2022; Zhao et al. 2023b). In comparison, carotenoids decline rapidly during the first 15 days of fermentation, though the magnitude of their decrease is smaller than that of chlorophylls (Guo et al. 2021).

Numerous studies have demonstrated that the addition of exogenous microorganisms altered the microbial community structure and function. For example, a synthetic community of two highly aluminum-resistant bacteria isolated from rice rhizosphere increased rice yield and alleviated soil acidification and Al toxicity by shifting the microbial community composition (Liu et al. 2023). In CTLs fermentation, inoculation of *Acinetobacter* sp. 1H8 and *Acinetobacter indicus* 3B2 altered the bacterial community structure and metabolic pathways, enhancing tobacco flavor quality and increasing the content of volatile flavor compounds such as solanone (Zheng et al. 2022b). In this study, high-throughput sequencing and comprehensive metabolomics analysis showed that additional strains shifted bacterial community and multispecies synergism regulated complex metabolic networks within the CTLs. When *C. thiooxydans* DS309 was added, the increased abundances of *Bacillus cytotoxicus* and *Bartonella refiksaydamii* were negatively correlated with the lipid molecule MP1491, which was directly associated with the change in PPO activity (Figs. 5 and 8). On the contrary, the higher abundance of *B. cytotoxicus* was negatively correlated with AAO activity, indicating that exogenous microorganisms may deepen the color of CTLs by accelerating lipid deterioration via their influence on polyphenol oxidation processes (Jiang et al. 2021). Moreover, declined abundances of *P. fulva* and *S. stutzeri* in all three bacteria-additional groups were positively correlated with the content of alkaloids MP553, suggesting the additional bacteria could enhance tobacco flavor, reduce irritation, and improve tobacco quality by decreasing alkaloids. In addition, despite the sulfonamide compound MP11566 was positively correlated with up-regulated abundance of *Staphylococcus nepalensis* in both the DS101 and DS309 groups (Fig. 8), the function related to flavor or color needs to be further investigated.

## Conclusion

In this study, we demonstrated the feasibility of influencing tobacco fermentation via the introduction of exogenous microorganisms with the aim of improving color of CTLs. Based on the correlation analyses of enzyme activity, bacterial community structure, and metabolite composition, it was revealed that the addition of exogenous strains *Sphingomonas parapaucimobilis* DS1, *Kosakonia cowanii* DS101, and *Comamonas thiooxydans* DS309 led to alterations in the abundances of various microorganisms and collaboratively regulated the color variations of CTLs by enhancing oxidation of polyphenols to brown quinones and oxidation of unsaturated fatty acids, resulting in membrane lipid peroxidation. This study puts forward a novel concept concerning the exogenous addition of microorganisms for the improvement of the color and quality of cigar wrapper.

## Electronic supplementary material

Below is the link to the electronic supplementary material.

Supplementary Material 1

Supplementary Material 2

## Data Availability

The datasets used and/or analysed during the current study are available from the corresponding author on reasonable request. DS1, JBPKAB000000000. https://www.ncbi.nlm.nih.gov/nuccore/JBPKAB000000000.1/ DS101, JBPKAC000000000. https://www.ncbi.nlm.nih.gov/nuccore/JBPKAC000000000. DS309, JBPKAD000000000. https://www.ncbi.nlm.nih.gov/nuccore/JBPKAD000000000.

## Abbreviations

CTLs: Cigar tobacco leaves
