# Supplementary figures and images for: Foliar microbiota confers deeper color of fermented cigar wrapper under additional fermented bacteria
Source: Bioresour Bioprocess. 2025 Jul 22;12(1):78. doi: 10.1186/s40643-025-00921-5 (PMC12283526; doi:10.1186/s40643-025-00921-5)

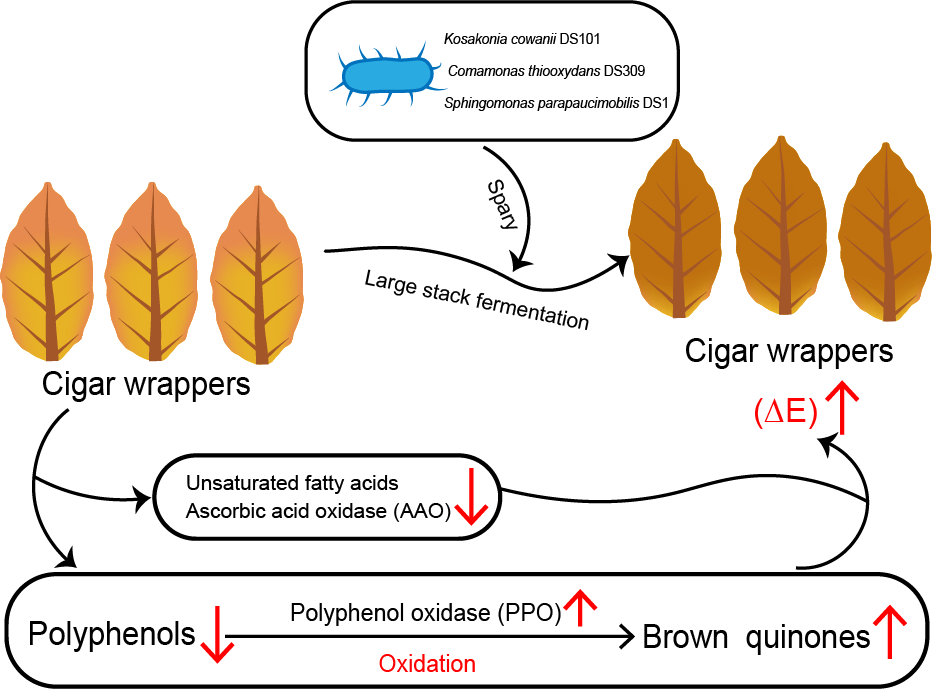

Supplement: Supplementary file 1 — Supplementary Material 1 [file 40643_2025_921_MOESM1_ESM.png]
