# Supplementary material for: Foliar microbiota confers deeper color of fermented cigar wrapper under additional fermented bacteria
Source: Bioresour Bioprocess. 2025 Jul 22;12(1):78. doi: 10.1186/s40643-025-00921-5 (PMC12283526; doi:10.1186/s40643-025-00921-5)
Supplement: Supplementary file 2 — Supplementary Material 2 [file 40643_2025_921_MOESM2_ESM.docx]

**Appendix A Supplementary data**

**Foliar microbiota confers deeper color of fermented cigar wrapper under additional fermented bacteria**

Qian Wu^a^, Xuanshuang Duan^a^, Dapeng Tang^b^, Rui Wang^c^, Fuqiang Li^b^, Jun Tan^b^, Honghua Chen^b^*, Shixue Zheng^a^*

^a^ National Key Laboratory of Agricultural Microbiology, College of Life Science and Technology, Huazhong Agricultural University, Wuhan, Hubei 430070, P. R. China

^b^ Enshi Branch, Hubei Tobacco Company, Enshi 445000, Hubei, P. R. China

^c^ Tobacco Research Institute of Hubei Province, Wuhan, Hubei 430033, P. R. China

* Corresponding authors

Shixue Zheng Email: [zhengsx@mail.hzau.edu.cn](mailto:zhengsx@mail.hzau.edu.cn) Phone/Fax: +86-27-87280670

Honghua Chen Email: 418539081@qq.com

## **Supplementary methods**

**Quality identification of cigar wrappers.**

The evaluation of the quality of the cigar wrappers at the end of the fermentation was done by experts from the cigar industry organised by the Cigar Tobacco Fermentation Factory in Laifeng County, Enshi City, Hubei Province, China, to evaluate the leaf identity, completeness and oil content of the cigar wrapper tobaccos.

**
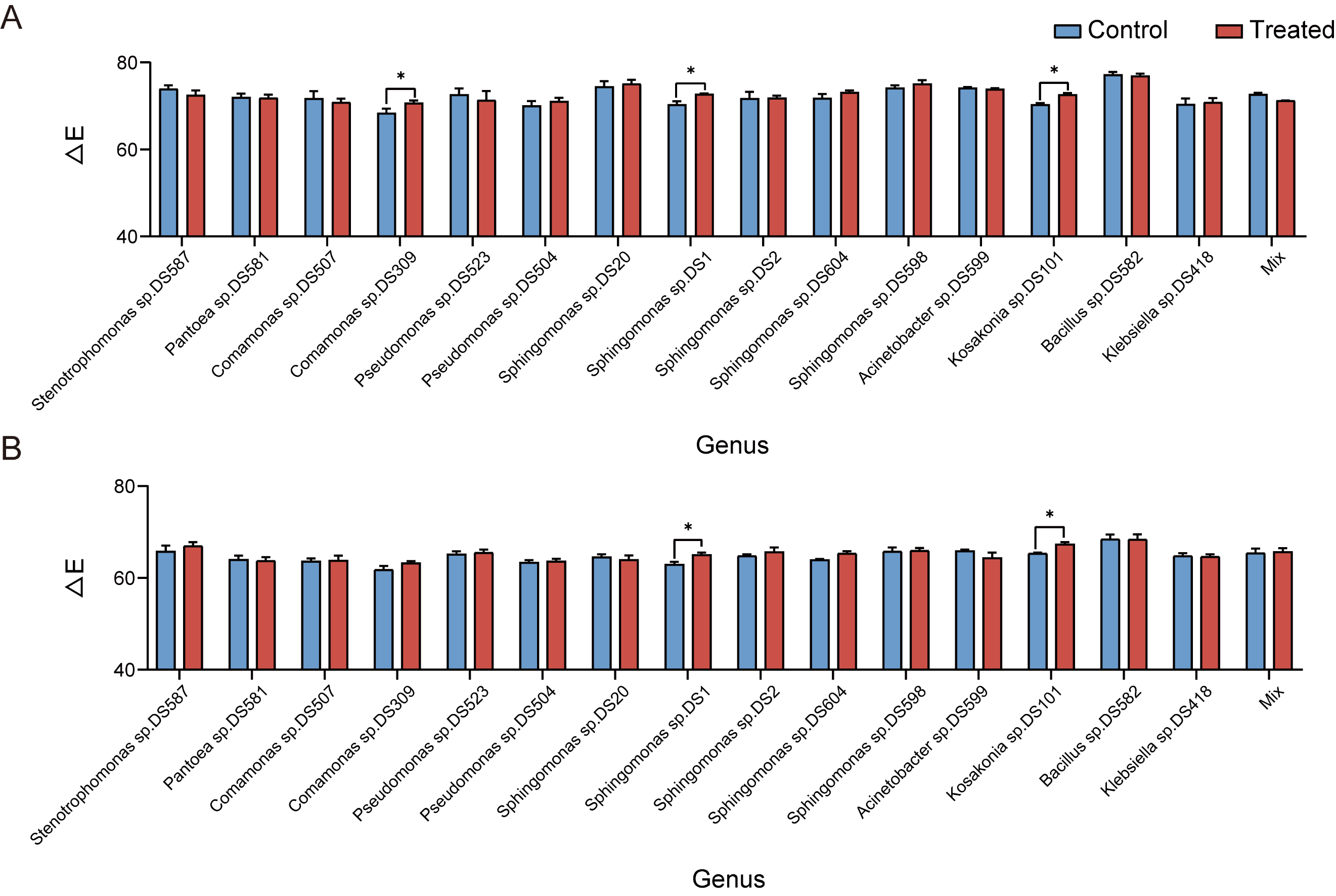
**

**Fig. S1 Total color difference value (ΔE) of the front or back side of cigar wrappers under half-leaf fermentation added with bacterial strains**

(A) ΔE of the front side of cigar tobacco leaves between treatment and control. (B) ΔE of the back side of cigar tobacco leaves between treatment and control.

**Table S1 Comparative analysis of** **ANI and dDDH based on genomes between strain DS1 and related type strains**

|  | DS1 | |
| --- | --- | --- |
|  | ANI values (%) | dDDH values (%) |
| *Sphingomonas parapaucimobilis* NBRC 15100 | 97.6 | 79.7 |
| *Sphingomonas yabuuchiae*  DSM *14562* | 91.35 | 43.8 |
| *Sphingomonas zeae* DSM *10049* | 88.37 | 35.4 |

**Table S2 Comparative analysis of ANI and dDDH based on the genomes between strain DS101 and related type strains**

|  | DS101 | |
| --- | --- | --- |
|  | ANI values (%) | dDDH values (%) |
| *Kosakonia cowanii* JCM 10956 | 96.9 | 72.2 |
| *Kosakonia sacchari* SP1 T5625 | 82.98 | 26.0 |
| *Kosakonia sacchari* CGMCC 1.12102 | 82.93 | 26.0 |

**Table S3 Comparative analysis of ANI and dDDH based on genomes between strain DS309 and related type strains**

|  | DS309 | |
| --- | --- | --- |
|  | ANI values (%) | dDDH values (%) |
| *Comamonas thiooxydans* DSM 17888 | 96.3 | 66.8 |
| *Comamonas testosteroni* ATCC 11996 | 94.4 | 56.9 |
| *Comamonas resistens* ZM22 | 84.5 | 28.7 |

**Table S4 Description and quality grading of cigar wrapper blades under different conditions**

| Treatment | Leaf Identity | Thinness | | Oil Content | Completeness |
| --- | --- | --- | --- | --- | --- |
| Control | Middle part of cigar tobacco | thin | Sufficient | | Relatively complete |
| *S. parapaucimobilis* DS1 | Middle part of cigar tobacco | thin | Sufficient | | Relatively complete |
| *K. cowanii* DS101 | Middle part of cigar tobacco | thin | Sufficient | | Relatively complete |
| *C. thiooxydans* DS309 | Middle part of cigar tobacco | thin | Sufficient | | Relatively complete |
